# Javamide-II Inhibits IL-6 without Significant Impact on TNF-alpha and IL-1beta in Macrophage-Like Cells

**DOI:** 10.3390/biomedicines8060138

**Published:** 2020-05-29

**Authors:** Jae B. Park, Renee Peters, Quynhchi Pham, Thomas T. Y. Wang

**Affiliations:** Diet, Genomics, and Immunology Laboratory, Bldg. 307C, Rm. 131, BHNRC, ARS, USDA, Beltsville, MD 20705, USA; renee.peters@usda.gov (R.P.); quynhchi.pham@usda.gov (Q.P.); tom.wang@usda.gov (T.T.Y.W.)

**Keywords:** Javamide-II (*N*-caffeoyltryptophan), Infection, COVID-19, IL-6 inhibition, p38/ATF-2, macrophage-like THP-1 cells

## Abstract

The main aim of this study is to find a therapeutic compound to inhibit IL-6, not TNF-alpha and IL-1beta, in macrophage-like cells, because the high-levels of IL-6 production by macrophages are reported to cause unfavorable outcomes under several disease conditions (e.g., autoimmune diseases, and acute viral infections, including COVID-19). In this study, the potential effects of javamide-II on IL-6, IL-1beta and TNF-alpha productions were determined using their ELISA kits in macrophage-like THP-1 cells. Western blots were also performed using the same cells, to determine its effects on signaling pathways (ERK, p38, JNK, c-Fos, ATF-2, c-Jun and NF-κB p65). At concentrations of 0.2–40 µM, javamide-II inhibited IL-6 production significantly in the THP-1 cells (IC_50_ of 0.8 µM) (*P* < 0.02). However, javamide-II did not inhibit IL-1beta or TNF-alpha productions much at the same concentrations. In addition, the treatment of javamide-II decreased the phosphorylation of p38 without significant effects on ERK and JNK phosphorylations in the THP-1 cells. Furthermore, the p38 inhibition, followed by the reduction of ATF-2 phosphorylation (not c-Fos, c-Jun or NF-κB p65), led to the suppression of IL-6 mRNA expression in the cells (*P* < 0.02). The data indicate that javamide-II may be a potent compound to inhibit IL-6 production via suppressing the p38 signal pathway, without significant effects on the productions of TNF-alpha and IL-1beta in macrophage-like THP-1 cells.

## 1. Introduction

Interleukin-6 (IL-6) is a pleiotropic cytokine that plays crucial roles in not only the immune functions but also a variety of biological processes, such as metabolism, bone homeostasis and cognitive functions [1,2,3,4,5,6,7,8]. Interleukin-6 is produced by numerous cells, including macrophages, monocytes, and T- and B-lymphocytes [1,2]. Especially, IL-6 is quickly produced from macrophages in response to infections (e.g., microbial, virus) and wounds, for protecting the host by stimulating immune reactions [2,3,4,5]. However, the high-levels of IL-6 production by macrophages are reported to have unfavorable consequences on inflammation, autoimmune diseases and other diseases [3,4,5,6,7,8,9]. Moreover, the dysfunction of IL-6 regulation is often found to be associated with the progression of several diseases, such as diabetes, liver diseases, rheumatoid arthritis and Crohn’s disease [3,4,5,6,7,8,9]. Furthermore, some reports suggest that perpetual elevation of IL-6 may even be related to the progression of acute viral infections, including COVID-19, and the inhibition of IL-6′s effects may have beneficial effects on mitigating the disease [10,11]. Therefore, the compounds that inhibit IL-6 have been relentlessly searched for in attenuating IL-6-related diseases [3,4,5,6]. In fact, several IL-6 inhibitors are now available, but most of them were reported to have undesirable side-effects or low efficacy [5]. In addition, these inhibitors often inhibit IL-6 with other inflammatory cytokines (e.g., TNF-alpha and IL-1beta), making it unfeasible to determine discrete outcomes of IL-6 selective inhibition in cells [5,6,7,8,9].

In our laboratory, we have screened/investigated therapeutic compounds able to inhibit inflammatory cytokines, including IL-6, for years. Recently, our and other studies suggested that coffee may contain several anti-inflammatory compounds, although the compounds responsible for the action have not been fully identified [12,13,14,15,16,17,18,19,20,21,22]. Javamide-I (*N*-coumaroyltryptophan) and javamide-II (*N*-caffeoyltryptophan) are tryptophan-derived phenolic amide compounds found in plant sources, including coffee [18,19,20,21,22]. Although javamide-I/-II contain a very similar chemical structure, our recent studies suggest that javamide-II and its analogues may have more potency than javamide-I in exerting several biological activities, including anti-inflammatory activity [18,19,20,21,22]. However, despite its potency, there is currently no information about the effect of javamide-II on IL-6 in macrophages which are critically participated in innate and adaptive immunity. Furthermore, its differential effects on other inflammatory cytokines, such as IL-1beta and TNF-alpha, have not been investigated as related to IL-6 inhibition, even though their inhibitions may have significant influences on biological outcomes of IL-6 inhibition. Therefore, in this paper, the potential effect of javamide-II on the expression of IL-6 cytokine was first investigated in PMA (phorbol 12-myristate 13-acetate)-differentiated macrophage-like THP-1 cells. Then, the inhibitory effects of javamide-II on TNF-alpha and IL-1 beta productions were comparatively examined with those on IL-6, in order to gauge its IL-6 selectivity [23,24,25,26,27]. Additionally, in this study, the effects of javamide-II on major signal transduction pathways (ERK (extracellular-signal-regulated kinase), JNK (c-Jun N-terminal kinase), p38) and associated transcriptional factors (ATF-2, c-Fos, c-Jun, and NF-κB p65) were also investigated to elucidate the underlying mechanism of IL-6 inhibition in the macrophage-like THP-1 cells.

## 2. Materials and Methods

### 2.1. Materials

Tryptophan, caffeic acid, 1,3-diisopropylcarbodiimide (DIC), dimethyl sulfoxide (DMSO) and other chemicals were purchased from Sigma Chemical Co. (St. Louis, MO, USA). Javamide-II was prepared as reported previously [19]. Briefly, caffeic acid (5 mmol) in DMSO (10 mL) was converted to the symmetrical anhydride with DIC (5 mmol), and then tryptophan (5 mmol) modified with phenylpropanol (5mmol) using a Fisher esterification method was added to the reaction mixture. The reaction mixture was incubated at room temperatures with a gentle stirring for 12 h. After that, the synthesized products were incubated under an alkaline condition (pH = 10) to remove phenylpropanol moiety, then javamide-II was purified by the HPLC (High Performance Liquid Chromatography (Waters, Milford, MA)) method as reported previously [19]. Briefly, Nova-Pak C18 (Waters, Milford, MA) was used as the stationary phase to purify synthesized javamide-II using a gradient condition; buffer A (50 mM NaH2PO4, pH 4.3) for 0–5 min, a linear change from buffer A to buffer B (40% acetonitril) for 5–40 min, and buffer B for 10 min at the flow rate of 1 mL/min. The javamide-II peak was monitored by CoulArray electrochemical detector (ESA, Chelmsford, MA, USA). The fractions with javamide-II were collected, heat-dried and dissolved in 10% ethanol (0.2 mL). The purified compound was also confirmed by NMR spectroscopic methods for this study. THP-1 cells were obtained from ATCC (Manassas, VA, USA). Antibodies for Western blots [Erk1/2 (catalog number 9102), phospho-Erk1/2 (catalog number 9101), p38 MAPK (catalog number 9212), phospho-p38 MAPK (catalog number 9211), JNK (catalog number 9252), phospho-JNK (catalog number 4668), ATF-2 (catalog number 9226), phospho-ATF-2 (catalog number 5112), c-Fos (catalog number 4384), phospho-c-Fos (catalog number 5348), c-Jun (catalog number 9165), phospho-c-Jun (catalog number 9164), NF-κB p65 (catalog number D14E12) and phospho-NF-κB p65 (catalog number 93H1)] were purchased from Cell Signaling Technology (Danvers, MA, USA).

### 2.2. Cell Culture

THP-1 cells were grown in RPMI 1640 medium with L-glutamine containing 10% FBS, 100 units/mL penicillin, and 100 units/mL streptomycin. The cells were maintained at 37 °C in a humidified atmosphere of 5% CO_2_. THP-1 cells were induced to differentiate into macrophage-like cells by incubating with phorbol 12-myristate 13-acetate (PMA; 25 ng/mL) for 48 h. The differentiated cells (1 × 10^6^ cells/well of 6-well plate) were treated with several concentrations of javamide-II (0–40 µM) followed by the treatment with lipopolysaccharide (LPS; 10 ng/mL) and incubated at specified times for each experiment.

### 2.3. Determination of the Productions of IL-6, IL-1beta and TNF-alpha Cytokines

The PMA-differentiated THP-1 cells were treated with javamide-II (0-40 µM) for 10 min, which was then followed by LPS treatment. After 18 h, the media samples were collected to determine the potential effects of javamide-II on the productions of IL-6, IL-1beta and TNF-alpha cytokines. In this cell culture model, LPS was used to stimulate the cells, because the LPS treatment induces macrophage-like THP-1 cells to the M1-like status cells which can produce several inflammatory cytokines including TNF-alpha, IL-1beta and IL-6 [26,27]. The concentrations of IL-6, IL-1beta and TNF-alpha in the media samples were respectively determined using Human IL-6, Human IL-1beta and TNF-alpha Quantikine ELISA (enzyme-linked immunosorbent assay) Kits from R&D systems (Minneapolis, MN, USA), according to manufacturer’s protocols.

### 2.4. Phosphorylation of ERK, p38 and JNK

For the Western blots of ERK, p38 and JNK, blot samples were prepared using the PMA-differentiated THP-1 cells treated with javamide-II (0, 10, 20, 40 µM) for 10 min, then followed by LPS treatment for 45 min. The Western blots were generated and blotted with phospho-ERK, ERK, phospho-p38, p38, JNK and phospho-JNK antibodies (Cell Signaling Technology, Danvers, MA, USA). For the blot, the amounts of protein in the samples were determined using Bio-Rad protein assay kit (Hercules, CA, USA), and ERK, p38 and JNK antibodies (Cell Signaling Technology, Danvers, MA, USA) were used as control samples for the blot.

### 2.5. Phosphorylation of c-Fos, ATF-2, c-Jun and NF-κB p65

For the Western blots of c-Fos, ATF-2, c-Jun and NF-κB p65, the THP-1 cells were treated with javamide-II as described above, and nuclear fractions for blot samples were prepared using a nuclear extraction kit (Cayman Chemical, MA, USA). The phosphorylation samples were generated with phospho-c-Fos, phospho-ATF-2, phospho-c-Jun and phospho-NF-κB p65 antibodies (Cell Signaling Technology, Danvers, MA, USA) and control samples were generated using c-Fos, ATF-2, c-Jun and NF-κB p65 antibodies (Cell Signaling Technology, Danvers, MA, USA).

### 2.6. Total RNA Isolation, Reverse Transcription Polymerase Chain Reaction ((RT)-PCR) and Gene Expression Analysis

Total RNA Isolation, reverse transcription (RT)-PCR and gene expression analysis were conducted as described previously [28]. Briefly, the THP-1 cells were treated with javamide-II, followed by LPS treatment for 4 h. After that, the samples were washed with PBS and TRIzol reagent was added for total RNA isolation. Affinity Script Multiple Temperature cDNA Synthesis kit (Agilent Technologies, Santa Clara, CA, USA) was used for cDNA synthesis and 1 µg of total RNA was used to reverse-transcribe mRNA to cDNA. Real-time PCR was performed on ViiA7 Real-Time PCR Detection System using 5 µL of cDNA by the TaqMan Universal Fast Master Mix according to the manufacturer’s protocol. TaqMan gene expression assay (Life Technologies, Carlsbad, CA, USA) was used to quantify gene expression levels of IL-6 (Hs00985639_m1), and human TATA box binding protein (TBP) (Hs00427620_m1) was used as a housekeeping gene. Relative expression levels were calculated using the ΔΔ*C*_t_ method as described previously [28].

### 2.7. Statistical Analysis

The IC_50_ and all statistical values were determined using the SigmaPlot 11.0 (Chicago, IL, USA). *P* values were calculated using one-way ANOVA (analysis of variance) with Holm–Sidak method, and *P* < 0.05 was considered as statistically significant. Data points in all figures were represented as the mean ± SD (n = more than 3).

## 3. Results

### 3.1. Effect of Javamide-II on the Production of IL-6 Cytokine

**Figure 1:**
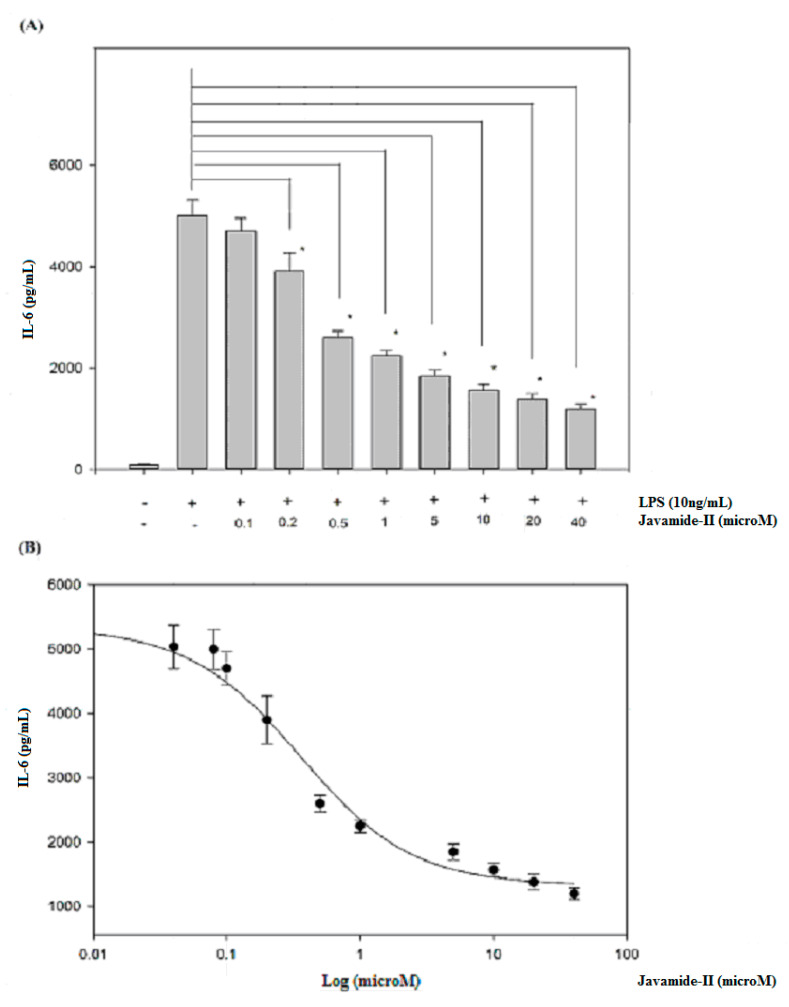
The effect of javamide-II on the expression of IL-6. (**A**) IL-6 production was determined at the concentrations of javamide-II (0, 0.1, 0.2, 0.5, 1, 5, 10, 20 and 40 µM) in LPS-treated PMA-differentiated THP-1 cells. The *P* value was calculated using one-way ANOVA with the Holm–Sidak method, and the asterisks (*) denote significant differences (*P* < 0.02) compared to the LPS control. (**B**) IC_50_ curve. Data points are shown as the means ± SD (*n* = 7).

To determine the potential effect of javamide-II on IL-6 production, the levels of IL-6 were determined in the media samples from the PMA-differentiated THP-1 cells treated with javamide-II (0–40 µM) followed by LPS treatment. As shown in Figure 1A, the treatment of LPS increased IL-6 production greatly. However, the production was significantly inhibited by the treatment of javamide-II in the THP-1 cells (Figure 1A) (*P* < 0.02). As shown in Figure 1A, all treatment groups except 0.1 µM showed significant reduction of IL-6 production compared to the LPS-only treatment. Furthermore, the inhibition was so strong that more than 20% and 40% of IL-6 production was inhibited, respectively, at 0.2 and 0.5 µM (Figure 1A). Due to its great potency, the IC_50_ value of javamide-II on IL-6 inhibition was determined, and found to be approximately 0.8 µM (Figure 1B).

### 3.2. Effects of Javamide-II on the Productions of TNF-alpha and IL-1beta Cytokines

**Figure 2:**
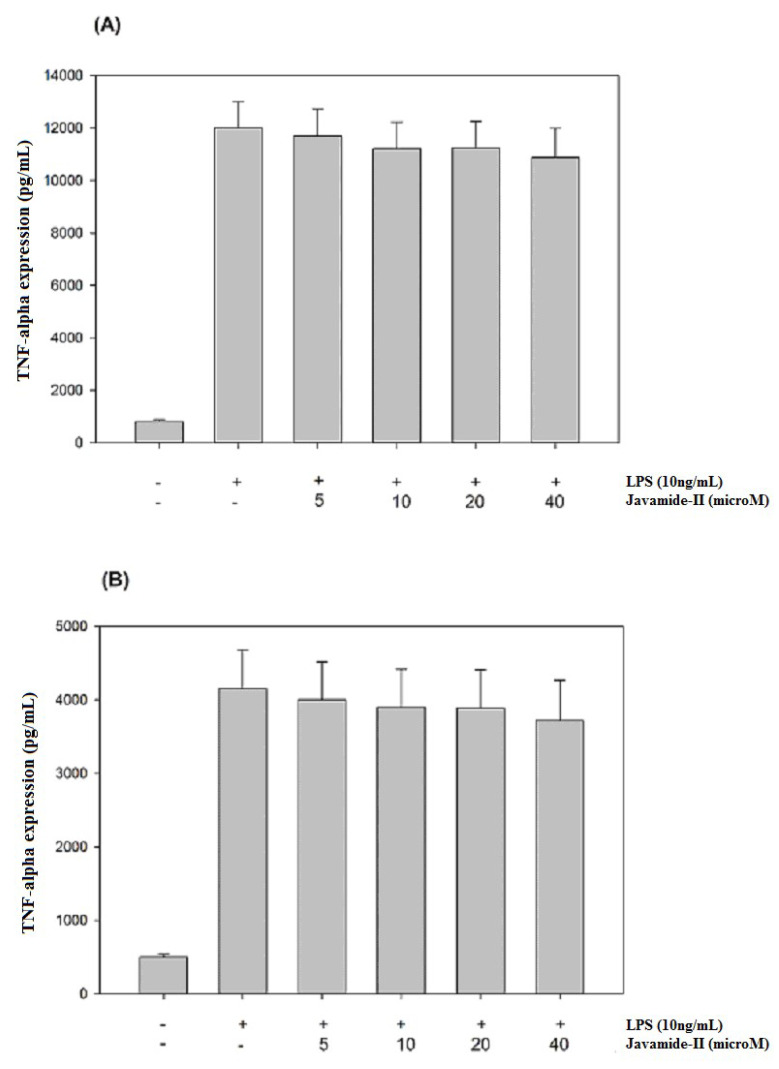
The effects of javamide-II on the expressions of TNF-alpha and IL-1beta. The productions of TNF-alpha (**A**) and IL-1beta (**B**) were determined at the concentrations of javamide-II (0, 5, 10, 20 and 40 µM) in LPS-treated PMA-differentiated THP-1 cells. Data points are shown as the means ± SD (*n* = 7).

Because javamide-II inhibited IL-6 significantly, and because several reports suggest that IL-6 expression may be closely associated with those of TNF-alpha and IL-1beta [23,24,25,26], the potential effects of javamide-II on the expressions of TNF-alpha and IL-1beta were investigated in the same THP-1 cells. As shown in Figure 2A,B, both the productions were significantly increased by LPS treatment. However, unlike IL-6, the productions of TNF-alpha and IL-1beta were not inhibited by the treatment of javamide-II (0–40 µM). These data suggest that the IL-6 inhibition by javamide-II may not coincide with IL-1beta and TNF-alpha inhibitions in the THP-1 cells. This finding is significantly different from the previous reports that the compounds that inhibit IL-6 (e.g., Takinib, JLU1124, SB-203580) can also inhibit TNF-alpha and IL-1beta [29,30,31,32]. These and our data clearly suggest that javamide-II may be different from those IL-6 inhibitors, and can suppress IL-6 expression in a way lacking IL-1beta and TNF-alpha inhibitions at concentrations lower than 40 µM (Figure 1 and Figure 2).

### 3.3. Effects of Javamide-II on the Phosphorylations of ERK, JNK and p38 MAPKs

**Figure 3:**
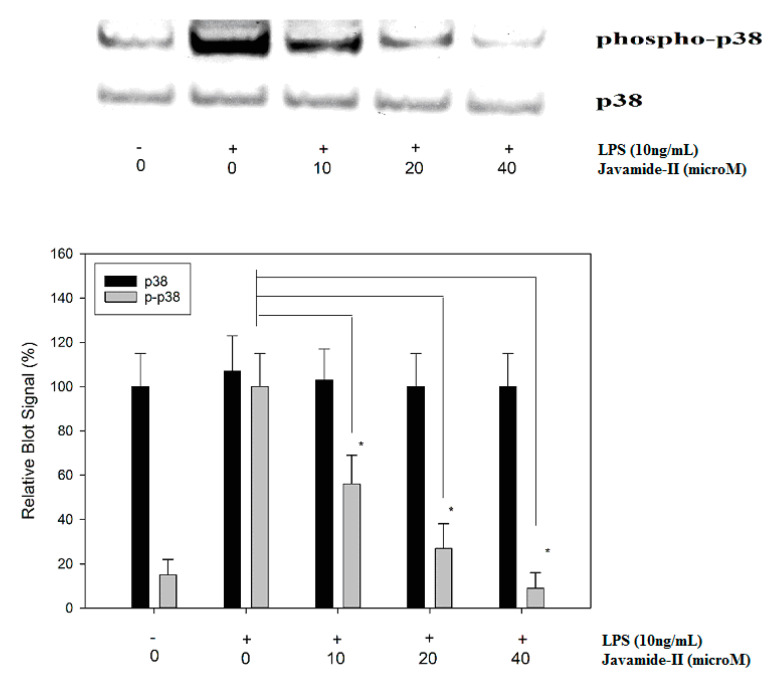
The effect of javamide-II on the phosphorylation of p38. The samples were prepared using PMA-differentiated THP-1 cells treated with javamide-II (0, 10, 20, 40 µM) followed by treatment with LPS for 45 min. Data points represent the means ± SD (*n* = 5). The *P* value was calculated using one-way ANOVA with the Holm–Sidak method, and the asterisks (*) indicate significant differences (*P* < 0.05) compared to the p38 control.

**Figure 4:**
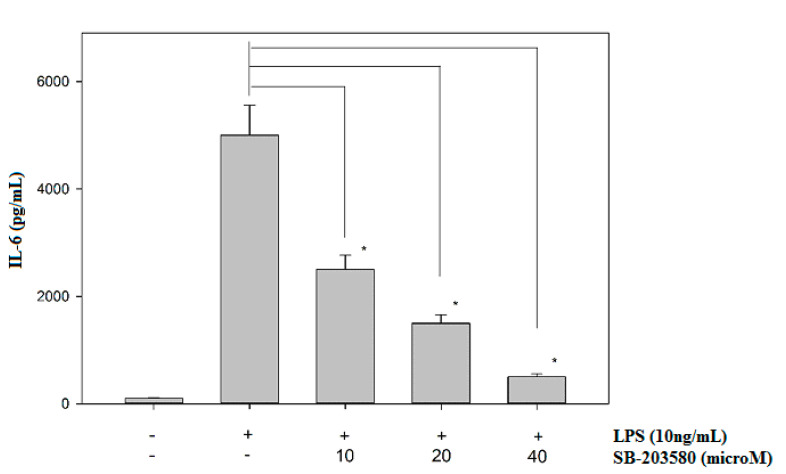
The effect of SB-203580 on the expression of IL-6. IL-6 production was determined at the concentrations of SB-203580 (0, 10, 20 and 40 µM) in LPS-treated, PMA-differentiated THP-1 cells. Data points are shown as the means ± SD (*n* = 5). The *P* value was calculated using one-way ANOVA with the Holm–Sidak method, and the asterisks (*) denote significant differences (*P* < 0.05) compared to the LPS control.

Mitogen-activated protein kinases (MAPKs; ERK, JNK and p38) are protein kinases activated by extracellular stimuli including LPS [29]. The treatment of LPS can increase ERK, JNK and p38 phosphorylations, and the activated MAPKs are greatly involved in the upregulation of several inflammatory cytokines, including IL-6 [30,31,32]. Therefore, potential effects of javamide-II on the phosphorylations of ERK, JNK and p38 were investigated to determine its influences on the MAP kinases. The treatment of javamide-II (10, 20, 40 µM) did not show any significant effect on the phosphorylation of ERK in the THP-1 cells (Data not shown here). Similarly, the treatment did not change the phosphorylation level of JNK in the cells either (Data not shown here). However, at the same range of concentration, javamide-II decreased the phosphorylation of p38 significantly in the THP-1 cells (Figure 3). These data suggest that javamide-II is likely to suppress p38 phosphorylation mostly in LPS-treated, PMA-differentiated THP-1 cells. To substantiate the involvement of p38 in the IL-6 production, SB-203580 (a p38 inhibitor) was tested and found to inhibit the expression of IL-6 in the same cells (Figure 4). These data suggest that javamide-II may inhibit the p38 pathway, which is one of the MAPK pathways closely associated with the production of inflammatory cytokines, including IL-6, in the cells [31,32].

### 3.4. Effects of Javamide-II on the Phosphorylations of ATF-2, c-Fos and c-Jun

**Figure 5:**
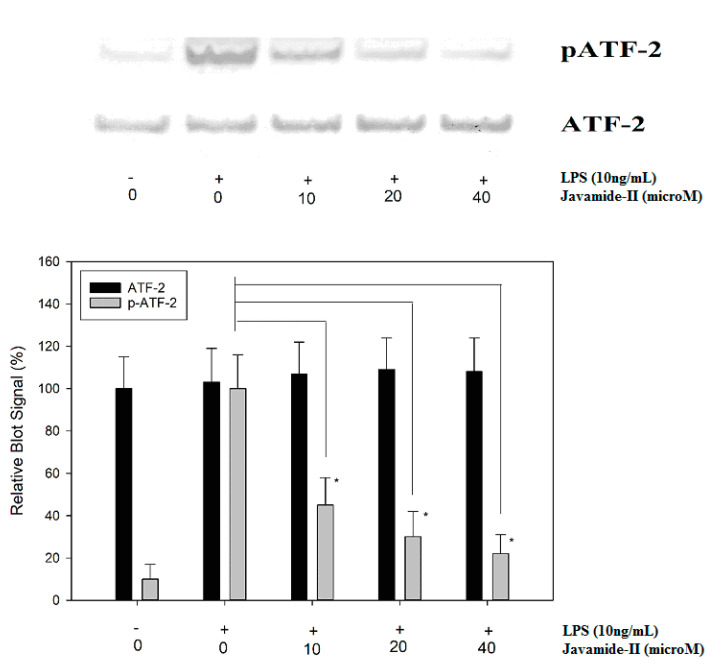
The effect of javamide-II on ATF-2 phosphorylation. PMA-differentiated THP-1 cells were treated with javamide-II (0, 10, 20, 40 µM) followed by treatment with LPS for 45 min, and the nuclear extract samples for blots were prepared as described in “Materials and Methods”. Data points represent the means ± SD (*n* = 5). The *P* value was calculated using one-way ANOVA with the Holm–Sidak method, and the asterisks (*) indicate significant differences (*P* < 0.05) compared to the ATF-2 controls.

In response to LPS treatment, IL-6 expression is induced significantly, but the expression was significantly inhibited by javamide-II, which could suppress p38 phosphorylation in the THP-1 cells. In fact, p38 can phosphorylate its down-stream signal transduction molecules, including ATF-2 (activating transcription factor-2), an important transcriptional factor involved in the production of several inflammatory cytokines [31,32,33,34]. Therefore, the effect of javamide-II on ATF-2 phosphorylation was investigated in LPS-treated THP-1 cells. As expected, the treatments of javamide-II (10, 20, 40 µM) inhibited the phosphorylation of ATF-2 in the cells (Figure 5). However, the treatment of javamide-II showed no significant effects on c-Fos and c-Jun phosphorylations, which are respectively modulated by ERK and JNK (Data not shown here). These data suggest that javamide-II may reduce the phosphorylation of ATF-2 via inhibiting p38, similarly to p38 inhibitors, which can reduce ATF-2 phosphorylation in the cells [32,35].

### 3.5. Effect of Javamide-II on NF-κB p65 Phosphorylation

**Figure 6:**
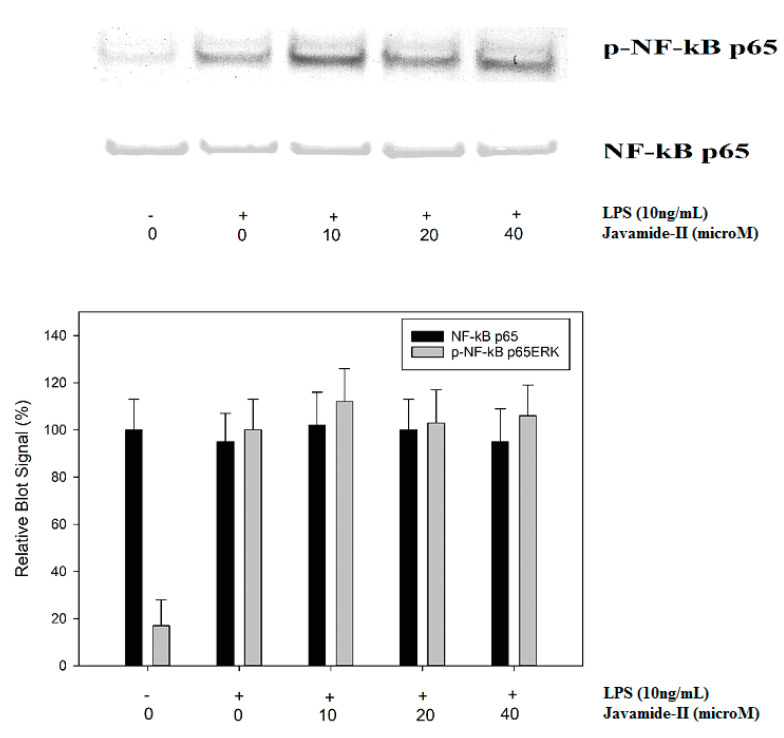
The effect of javamide-II on p65 NF-κB phosphorylation PMA-differentiated THP-1 cells were treated with javamide-II (0, 10, 20, 40 µM) followed by treatment with LPS for 45 min, and the nuclear extract samples for blots were prepared as described in “Materials and Methods”.

Nuclear factor-kappa B (NF-κB) is another key transcriptional factor profoundly involved in the expression of a slew of inflammatory cytokines, especially TNF-alpha and IL-1beta [35,36,37]. In THP-1 cells, it is reported that LPS treatment can activate the I-kB-alpha kinase complex (IKK), then the activated IKK can phosphorylate I-kB and NF-κB p65, leading to nuclear localization and transactivation of several downstream genes [36,37,38]. Therefore, the potential effect of javamide-II on NF-κB p65 phosphorylation was investigated in the THP-1 cells. As expected, the LPS treatment increased the phosphorylation of NF-κB p65, but the phosphorylation was not significantly inhibited by javamide-II. These data indicate that javamide-II may have no significant effect on the NF-κB pathway (Figure 6), and the production of IL-6 may be inhibited by javamide-II mainly via the suppression of the p38/ATF-2 signal pathway in the THP-1 cells.

### 3.6. Effect of Javamide-II on the Production of IL-6 mRNA

**Figure 7:**
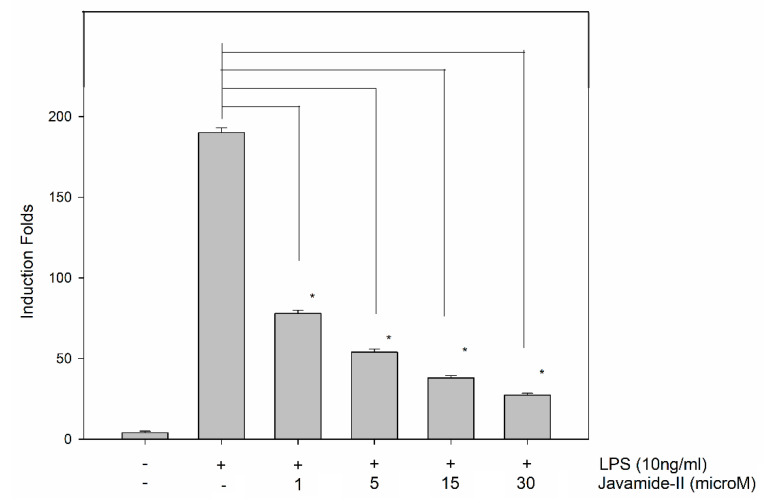
The effect of javamide-II on IL-6 mRNA. PMA-differentiated THP-1 cells were treated with javamide-II (0. 1, 5, 15, 30 µM) followed by treatment with LPS for 4 h as described in “Materials and Methods”. After 4 h, total RNA was isolated and gene expression was determined using (RT)-PCR. Data are expressed as mean ± SD (*n* = 3). The asterisks (*) indicate significant difference from vehicle control (*P* < 0.002).

Because javamide-II showed inhibitory effects on p38/ATF-2 phosphorylation, its potential effect on IL-6 mRNA was investigated in the THP-1 cells in order to assess the impact of the p38/ATF-2 inhibition on IL-6 gene expression. As expected, the treatment of LPS led to significant induction of IL-6 mRNA levels in the cells, but the up-regulated IL-6 mRNA production was significantly inhibited by javamide-II in a concentration-dependent manner (Figure 7). In fact, all treatment groups demonstrated significant reduction of IL-6 mRNA expression against the LPS-only treatment (Figure 7). These data suggest that the p38/ATF-2 inhibition by javamide-II may lead to the greatest suppression of IL-6 mRNA expression, because javamide-II may have little effect on the phosphorylations of ERK, JNK, c-Fos, c-Jun and NF-κB p65. These data clearly suggest that javamide-II can inhibit not only IL-6 protein production, but also its mRNA in the THP-1 cell, and the inhibition of p38/ATF-2 may be behind the suppression IL-6 mRNA. Altogether, these data suggest that javamide-II may be a compound that inhibits IL-6 production selectively via suppressing the phosphorylation of p38/ATF-2, without significant effects on TNF-alpha and IL-1beta productions in PMA-differentiated THP-1 cells.

## 4. Discussion

IL-6 is a cytokine involved in diverse physiological processes, such as T-cell activation, induction of immunoglobulin secretion, hepatic acute phase protein production and metabolic functions [3,4,5,6,7,8,9,10,11]. Particularly, IL-6 cytokine is critically involved in the progression of several inflammation-related/autoimmune diseases, such as rheumatoid arthritis (RA), liver and inflammatory bowel disease, and acute viral infection [5,6,10,11]. Several reports also suggest that the persistent activation of the IL-6 signaling pathway may play a critical role in the progression of the diseases mentioned above [39,40,41,42,43]. Furthermore, recent COVID-19 studies indicate that high levels of IL-6 may be associated with COVID-19 infection, and that the inhibition of IL-6 may be beneficial in extenuating the disease [11,43]. For these reasons, therapeutic agents able to control IL-6 expression have been explored for years, to treat the progression of IL-6-related diseases [40,41,42,43]. In our laboratory, potent compounds that inhibit inflammatory cytokines, including IL-6, have been investigated for more than 10 years, and javamide-II is one of compounds of interest in our recent research. In fact, this compound is a tryptophan-derived phenolic amide compound found in plant sources including coffee [8]. Recently, our studies suggested that javamide-II and analogues may have several biological activities, including anti-inflammatory activity [18,19,20,21]. However, there is currently no information about potential effects of javamide-II on the expressions of IL-6, IL-1beta and TNF-alpha cytokines in macrophages. To investigate its potential effects on the cytokines from macrophages, a LPS-treated, PMA-differentiated THP-1 cell culture model was used in this study, because the LPS treatment can induce macrophage-like PMA-differentiated THP-1 cells to the M1-like status cells, which are often found under several inflammatory disease conditions, including RA, liver disease and viral infection, and produce several inflammatory cytokines (e.g., IL-6, IL-1beta and TNF-alpha) [26,27,39,40,41,42,43,44].

As shown in Figure 1 and Figure 2, javamide-II was found to inhibit the production of IL-6 without significant influence on TNF-alpha and IL-1beta. These data suggest that javamide-II may inhibit IL-6 selectively in a way deprived of much of the IL-1beta and TNF-alpha inhibitions. Furthermore, the data showed that the IL-6 inhibition went through the suppression of the phosphorylation of p38 MAPK (Figure 3). p38 MAPK is a serine/threonine kinase, widely expressed in cells (e.g., endothelial, immune and inflammatory cells), and is one of MAPK kinases involved in producing pro-inflammatory cytokines [30,31,32]. Interestingly, several studies suggest that p38 may be involved in several inflammatory diseases (e.g., RA, liver and other diseases) [44,45,46,47,48,49,50] and the inhibition of p38 is likely to be a promising therapeutic option for treating these diseases [49,50,51]. Currently, several p38 inhibitors are available for use, but most of them are not truly selective IL-6 inhibitors, making it unfeasible to assess the pathophysiological contributions from lone IL-6 inhibition. Our data suggest that javamide-II may be a potential candidate compound to suppress IL-6 selectively via p38 inhibition (Figure 3). The data also showed that the p38 inhibition was followed by the reduction of ATF-2 phosphorylation (Figure 5), and p38/ATF-2 inhibition could lead to the inhibition of mRNA IL-6 expression (Figure 7), suggesting that the suppression of the p38 signal pathway may be a key pathway leading to the inhibition of IL-6 production in macrophage-like THP-1 cells. As indicated above, the selective cytokine inhibition is likely to rely on a delicate control balance of complicated signal transduction pathways. In fact, our study showed the inefficacy of javamide-II on the NF-κB pathway (Figure 6) critically involved in the expression of TNF-alpha and IL-1beta [35,36,37]. Therefore, it is cautiously supposed that the differential control of these signal transduction pathways may be behind the selective IL-6 inhibition in the macrophage-like THP-1 cells.

Also, related to the p38 signal inhibition, javamide-II may not inhibit p38/ATF-2 phosphorylation in every cell, as demonstrated by our other study [21]. For instance, when the signal pathway inhibition by javamide-II is compared in macrophage-like THP-1 and lymphocytic Jurkat cells, we found that the inhibition pattern was different in these two cells, with the inhibition of p38 phosphorylation in the macrophage-like THP1 cells, but the inhibition of ERK phosphorylation in the lymphocytic Jurkat cells [21]. This implies that javamide-II may exert cell-specific actions by delivering disparate effects to each type of cell, or at least these two cells (e.g., macrophages and lymphocytes). Nonetheless, in macrophage-like THP-1 cells, javamide-II showed great potency in suppressing IL-6 (IC_50_ of 0.8 µM) (Figure 1A,B). Unfortunately, it is currently impractical to compare the efficacy of javamide-II with other IL-6 inhibitors, because there is no true IL-6 selective inhibitor available. However, during the study, javamide-II was compared with SB-203580 as related to IL-6 inhibition, and javamide-II was found to be more effective in IL-6 inhibition than SB-203580, especially at concentrations of less than 10 µM (data shown here). Furthermore, our data suggest that the javamide-II may be better in inhibiting IL-6 selectively than SB-203580, because SB-203580 often inhibits IL-6, IL-1beta and TNF-alpha altogether in the cells [32]. Although there are some limitations in using cell lines, the use of proper cell lines often provides a lot of advantages (e.g., homogenous characters, excellent usability and speedy screen-ability). Because the main aim of this study is to find a compound to inhibit IL-6 preferably, the macrophage-like THP-1 cell line serves most of our purpose. Altogether, the data of this study suggest that javamide-II may be a potent compound, which may be used as a candidate molecule to inhibit IL-6 selectively in the macrophage-like cells.
